# Post-Traumatic Stress Disorder (PTSD) in Sexually Abused Children and Educational Status in Kenya: A Longitudinal Study

**DOI:** 10.4172/2375-4494.1000357

**Published:** 2017-09-28

**Authors:** Teresia Mutavi, Muthoni Mathai, Anne Obondo

**Affiliations:** Department of Psychiatry, School of Medicine, College of Health Sciences, University of Nairobi, Nairobi, Kenya

**Keywords:** Post-traumatic stress disorder, Child sexual abuse, Educational status, Kenya

## Abstract

Children who experience sexual abuse often meet the criteria of Post-Traumatic Stress Disorder and other psychiatric disorders. This article examines Post-Traumatic Stress Disorder and their educational status among children who have been sexually abused and its effects on the children’s educational status. The study was carried out between June 2015 and July 2016. The study adopted a longitudinal study design. The study was conducted at Kenyatta National Teaching and Referral Hospital and Nairobi Women’s Hospitals in Kenya. The children who had experienced sexual abuse and their parents/legal guardians were followed up for a period of one year after every four months interval. One hundred and ninety one children who had experienced sexual abuse and their parents/legal guardians were invited to participate in the study. Findings indicate that the children continued to experience PTSD one year after the sexual abuse incidence. PTSD was associated with the length of time taken to receive medical attention (p<0.005). Children with partial PTSD who had experienced sexual abuse were 2 times more likely to perform above average than children with full PTSD, OR=2.1 [95% CI of OR 1.2–3.8], p=0.01. Children who experience sexual abuse have negative mental health outcomes. These outcomes have detrimental effects to the normal development of children and educational status. There is need to screen for PTSD and offer psychosocial support and follow up to children who have been sexual abuse.

## Introduction

Post-Traumatic Stress Disorder (PTSD) refers to certain enduring psychological symptoms that occur in reaction to highly distressing, physically and psychologically disruptive events [1,2]. Sexual abuse against children is a psychological, Physical, social, psychiatric, educational and public health concern whose outcomes are not only detrimental to the health of children, but also, the health of their families and societies [3–5]. Children who experience sexual abuse often meet the criteria of PTSD and other psychiatric disorders [6,7]. Studies done on sexual abuse against children shows a relationship between sexual abuse and trauma symptoms of PTSD [8]. While males are more exposed to events that could be potentially traumatic such as accidents, assault or disaster, females particularly adolescents and young adults females are more likely to experience sexual abuse with the females to male ratio in the prevalence of PTSD approximately being 3:1 and females reporting higher levels of re-experiencing, avoidance and arousal [9,10]. Numbing of responsiveness is an instinctive baseline function among young children subjected to sexual abuse [11]. Sexually abused children in particular, have been known to react as if the approach of a stranger were precipitating a repeat attack [11]. PTSD in children is different from the adult disorder; children tend to re-experience the event through play or drawing and exhibiting nightmares involving monsters rather than the traumatic event [12]. A study on children with histories of sexual abuse in Australia found that the children significantly had higher levels of trauma-related symptomatology [13]. Another study in Denmark among sexual abuse survivors found that 94% of them met the criteria of re-experiencing, 99% met the criteria of arousal type and 90% met the criteria of avoidance while the prevalence of PTSD was found to be 80% [14]. Children suffering from PTSD were found to have lower rates of improvement when living in homes with high levels of conflict and where interpersonal violence was present [15]. When PTSD among male victims of sexual abuse is left untreated, it could lead to serious emotional and behavioural problems, including more incidents of criminal activities and violence later in life [16]. Not all children who are sexually abused have poor psychological outcomes [17]. Factors, such as spirituality, family and peer support, attachment style, hardiness or resiliency, as well as some coping strategies impacted on the degree of recovery among the children [18]. In comparing children with low perceived social support, with those with high perceived social support, a study in South Africa found out that those with high perceived social support demonstrated significantly lower levels of PTSD symptoms after both low and high levels of trauma exposure [19]. In another study done in Kenya on traumatic experiences among secondary school students found out that 85.3% of the students had full and partial symptoms of PTSD and 33% had experienced sexual abuse [20]. In yet another study in Kenya found out that the prevalence of PTSD among 61 sexually abused children was 45% and the prevalence of psychiatric morbidities was 69% [21]. Similarly another study in Kenya found out that among 149 sexually abused children the prevalence rate of PTSD was at 50% [22]. In the same study, PTSD was found to be associated with the degree of physical or verbal abuse during sexual abuse, injuries during the assault and parent-child relationships. In Kenya, there is currently paucity of literature on PTSD and educational status however studies indicate that in the US, the youth exposed to repeated community and family violence reported less positive school engagement and high psychological symptoms such as PTSD [23]. In addition, adolescents in the US exposed to community violence and trauma, were vulnerable to a cascade of events including psychological symptoms and decreased connectedness to school. He concluded that community violence and trauma ultimately led to overall poor academic achievement [24]. Untreated mental health disorders have significant and harmful impact on children, with strong evidence that academic learning is impeded significantly or prohibited entirely when youth suffer from mental health concerns such as PTSD [25]. This study sought to answer the question: Is PTSD and school status associated with sexual abuse of children in Kenya. Overall, there is need for a better understanding of psychosocial outcomes associated with sexually abused children, as well as the provision of psychosocial services for them. There is growing magnitude of sexual abuse against children in Kenya which translates into negative psychosocial outcomes such as PTSD and poor educational status. This study sought to determine the incidence of PTSD and educational status among children who have experienced sexual abuse in Kenya and follow up on the children for a period of one year.

## Methodology

**Figure 1:**
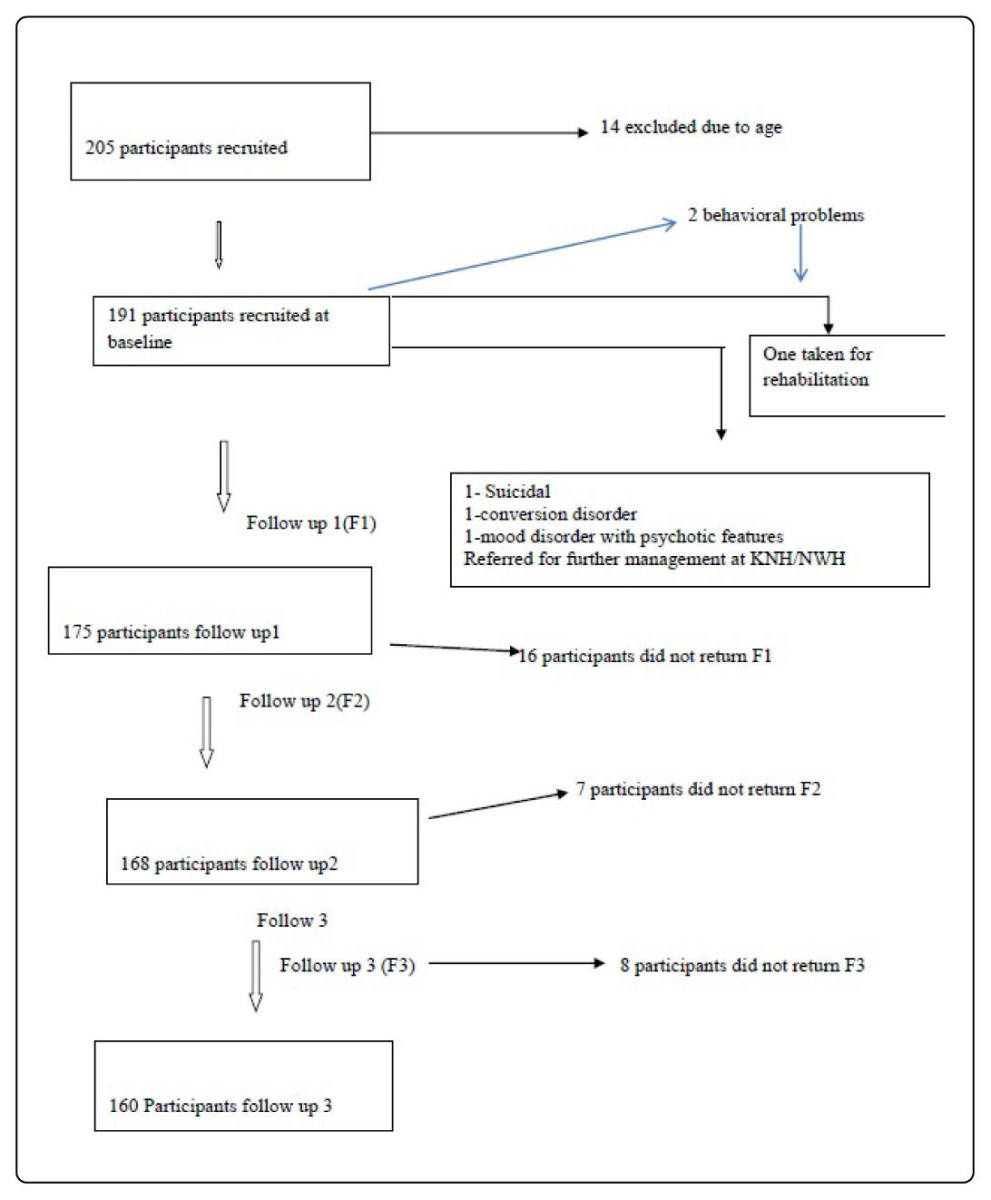
The flowchart depicting sample collection from baseline to follow up 3.

This paper is based on data of a longitudinal study design with data collected from June 2015 to July 2016 and follow-ups after every four-month interval at the two most specialized Gender Based Violence Recovery Centres (GBVRC) in Nairobi, the Kenyatta National Teaching and Referral Hospital (government) and Nairobi Women’s Hospital (private). Ethical and scientific research approval was obtained through the Kenyatta National Hospital/University of Nairobi Institutional Review Board. The study population included children aged 6½–17½ years who had experienced sexual abuse and their parents and or their legal guardians. The parents/legal guardians gave informed consent and the children gave assent to participate. Children with severe learning disabilities and those whose parents did not consent to participate in the study were excluded from participating in this study. To determine the sample size required to estimate the true incidence rate within a relative precision of 15% with 95% confidence level a minimum of 171 sexually abused children needed to be followed up with ε=0.15 and z=1.96 Lemeshow et al. [26]. A sample size of 191 with an assumed attrition rate of 20% was used. For this study, random sampling technique was used to recruit the parents/legal guardians of the children from the children’s files one month after the sexual abuse incidence (Figure 1).

By the time of recruitment, the children had been put on medical (post exposure prophylaxis against HIV and contraceptive prophylaxis), treatment of physical injuries and psychosocial care in the hospitals and referred to legal service providers; a standard prophylactic and therapeutic treatment provided at the GBVR centres in both hospitals. The study participants who after recruitment to the study showed the need for treatment were referred to physicians at the two centres. A locally designed questionnaire that aimed to capture the socio-demographic characteristics and sexual abuse profile was administered to the children and their parents or legal guardians by the researchers. The Child PTSD Symptom Scale (CPSS) was also administered to the children [27]. To meet the full PTSD according to the DSM-IV criteria including re-experiencing, avoidance, numbing and hyper-arousal had to be elicited in all the study participants. Foa et al. [27] found that CPSS was a reliable and valid screening tool for PTSD symptoms among children and adolescent with internal consistency ranging from 0.70 to 0.89 for the total and subscales symptom scores. Test-retest reliability was good to excellent (0.84 for the total score, .85 for re-experiencing, 0.63 for avoidance and 0.76 for hyper-arousal). Convergent validity was high. The CPSS correlated .80 with the Child Posttraumatic Stress Reaction Index [28] and the Child’s PTSD checklist which have been used in Kenya [20]. The CPSS was standardised in California with 75 school children approximately two years after the 1994 Northridge Californian Earthquake. The scale incorporates developmentally appropriate language which was translated into Kiswahili, back translated into English, and discrepancies were corrected by consensus. Educational status was assessed by checking the end of term school reports of each child. Completed questionnaires were safely and securely stored prior to entry into computer excel sheets for analysis. Descriptive and inferential statistics were conducted using SPSS Version 21. The level of statistical significance was set at 0.05 (p<0.05) with a 95% confidence level. Bivariate and multivariate logistic regression analyses were done to determine associations among variables.

## Results

A total of 191 study participants were recruited into the study. Four months later during follow up in this longitudinal study, there was an attrition rate of 8.4% of the study population (N=175) due to change of residence, contacts and non-response to calls. The attrition rate of the study population reached 15.7% (N=160) at 8 months follow up and it was 16.0% at the final follow 12 months later. Of the 191 recruited 23 (12.0%) were males and 168 (88.0%) were females, a male: female ratio of 1:7. The mean and median was 13. The youngest was 7 and the oldest were 17 years, respectively. Over two-thirds of the study participants 67 (35.1%) were aged between 13–15 years, 47 (24.6%) were aged between the age of 16–17 years, 44 (23%) were aged 10–12 years and 33 (17.3%) were aged between 7–9 years. Over three quarters (3/4) of the study participants 144 (75.4%) had both parents alive, 33 (17.3%) had mothers only, 8 (4.2%) had fathers only and 6 (3.1%) were parentless. Most of the parents 138 (72.2%) were married, 33 (17.3%) separated, 4 (2.1%) divorced, 16 (8.4%) Single. 58 (30.4%) of the parents/legal guardians earned less than Ksh100 a day, 71(37.2%) earned Ksh100 a day, 56(29.3%) earned more than Ksh100 while 6 (3.1%) earned more than ksh200 a day, implying that they all earned no more than $1 per day for their livelihoods (Tables 1–5).

The incidence of full PTSD was 182 (95.3%), while partial PTSD was 9 (4.7%) a month after the sexual abuse. At follow up 1, 166 (95%) had full PTSD and 9 (5%) had partial PTSD. At follow up 2, 102 (60.7%) had full PTSD and 66 (39.3%) had partial PTSD. At follow up 3, 96 (60%) had full PTSD and 64 (40%) had partial PTSD.

Educational status mean score improved over time from 249 marks out of 500 at baseline, 271 marks out of 500 during the first follow up, 290 marks out of 500 during the second follow up but slightly dropped to 288 marks out of 500 during the third follow-up. Full PTSD was significantly associated with below average performance (p<0.0001). Sexually abused children with partial PTSD were 2 times more likely to perform above average than children with full PTSD OR=2.1 [95% CI of OR 1.2–3.8], p=0.01.

## Discussion

This study investigated the incidence of PTSD among children who had been sexual abused. The results have shown very high levels of PTSD which continued even up to one year follow up. These findings are consistent with study finding found in Kenya on traumatic events and PTSD outcomes among children which found that 85.3% developed PTSD [20]. Similar findings were also noted in Denmark in a study among sexual abuse survivors [14]. In this study children who received medical care early were significantly more likely to have PTSD. This is surprising finding and serious finding given the requirement that cases of rape report within 72 hours to receive post exposure prophylaxis against HIV and contraceptive prophylaxis, Other studies on PTSD done among children did not report this, but again did not look for this factor. This raises the question of retraumatization. Studies have shown that, previous popular method of acute psychological treatment and debriefing was found to be responsible for retraumatization and has been discredited. There is then a need to review the treatment methods in these health facilities.

The findings of this study that there was no significant difference in age, both young and older children experience PTSD after exposure to a traumatic event was similar to Ndetei et al. [20] and Harder et al. [29]. However, this differs significantly with study by Giannopoulou et al. [30] who found that younger children suffer more from PTSD compared to older children and adolescents. This could be attributed to the difference on the type of traumas that the children in the different studies were exposed to in Kenya.

The findings of this study that sexually abused children developed PTSD and had still PTSD even after one year follow up corresponds to other studies [31]. In their study sexually abused children in Germany had four times the odds of developing PTSD compared with the general population of children experienced other forms of maltreatment and the children still were diagnosable for PTSD at follow up 3 years after the sexual abuse incidence. The likelihood of developing PTSD was greater than the likelihood of developing any other outcomes investigated such as depression [32]. Another study found that one in 4 sexually abused children developed PTSD after exposure to sexual abuse and interpersonal trauma [33]. In other studies, PTSD was maintained over time due to trauma related shame [34,35]. Prolonged psychological trauma was also found in Kenya among children who had experienced sexual abuse [36]. In their study they noted that sexual abuse on children is accompanied by deeper prolonged psychological trauma and that PTSD in sexually abused children was more traumatic psychologically than ordinary trauma.

The current study finding on sexually abused children experiencing difficulties in educational status compares to study findings by Albano et al. [37]. Students with mental disorders are more likely to doubt their academic competence thus interfering with their ability to complete assignments. This can lead to underachievement, problems related to concentrating in school work and doing homework. The time these students spend academically engaged is also affected by the incidence of sexual abuse against children as the PTSD and depression triggers overwhelming thoughts which impede their concentrations. This explain the reason why the children were performing poorly immediately after the incidence of sexual abuse against children.

## Conclusion and Recommendations

The study concludes that sexually abused children included in this study had mental health problems as measured by Child PTSD Symptom Scale. Girls were more at risk of experiencing sexual abuse than boys. The findings of this study suggest the need for psychosocial support for children who sought services at the Nairobi Women’s and Kenyatta National Hospitals, as well as other children. There is also need to relook at the methods of delivery of psychosocial support in order to prevent retraumatization to children who report early after incidence of sexual abuse. The provision of a comprehensive health care by a multi-disciplinary team, including psychiatrists and social workers is warranted. Moreover, there is the need to (a) enhance parenting protective skills and (b) minimize risk factors that could lead to poor mental health outcomes among children after the incidence of sexual abuse.

## Figures and Tables

**Table 1:** Incidence of PTSD from June 2015 to July 2016.

PTSD severity								
	Baseline N	%	Follow up 1	%	Follow up 2	%	Follow up 3	%
Full PTSD	182	95.3	166	95	102	60.7	96	60
Partial PTSD	9	4.7	9	5	66	39.3	64	40
	**191**	**100**	**175**	**100**	**168**	**100**	**160**	**100**

**Table 2:** Association between PTSD and socio demographic characteristics of the children at baseline (Bivariate Analysis).

	PTSD				
	Partial PTSD		Full PTSD		
	N=9		N=182		p-value
**Gender**	**N**	**(%)**	**N**	**(%)**	
Male	1	(4.3)	22	(95.7)	**0.93**
Female	8	(4.8)	160	(95.2)	
**Age group**					
07–09	2	(6.1)	31	(93.9)	**0.735**
10–12	3	(6.8)	41	(93.2)	
13–15	3	(4.5)	64	(95.5)	
16–17	1	(2.1)	46	(97.9)	
**Attending school**					
Yes	8	(4.3)	177	(95.7)	**0.055**
No	1	(25.0)	3	(75.0)	
**School level**					
Primary	9	(4.2)	158	(95.8)	**0.36**
Secondary	0	(.0)	19	(100.0)	
**Have parents**					
Both parents	7	(4.9)	137	(95.1)	**0.662**
Only Mother	1	(3.0)	32	(97.0)	
Only Father	1	(12.5)	7	(87.5)	
None	0	(.0)	6	(100.0)	
**Parents marital status**					
Married	5	(3.7)	131	(96.3)	**0.425**
Separated	2	(6.1)	31	(93.9)	
Divorced	0	(.0)	6	(100.0)	
Single	2	(12.5)	14	(87.5)	
**Who do you live with**					
Neighbours	0	(.0)	0	(.0)	**0.010**
Good Samaritan	1	(50.0)	1	(50.0)	
Care Giver	5	(3.1)	156	(96.9)	
Guardian	3	(12.5)	25	(87.5)	

PTSD in the sexually abused children was associated with the person the children lived with (p=0.01) at baseline.

**Table 3a:** Association between PTSD and sexual abuse profile at baseline (Bivariate Analysis).

	PTSD				
	Partial PTSD N=9		Full PTSD N=182		
	N	(%)	N	(%)	p-value
**Relationship to perpetrator**					
Stranger	6	(7.9)	70	(92.1)	**0.347**
Acquaintance	2	(2.0)	99	(98.0)	
Non parental care giver	0	(.0)	3	(100.0)	
Biological parent	1	(11.1)	10	(88.9)	
**Perpetrators acts**					
Vagina anal penetration	7	(4.1)	163	(95.9)	**0.167**
Touching the genitals	2	(18.2)	9	(81.8)	
**What perpetrator made victim to do**					
Nothing	2	(6.7)	14	(93.3)	**<0.0001**
Touching genitals	7	(4.1)	165	(95.9)	
Oral copulation	0	(.0)	3	(100.0)	
**Frequency of abuse**					
Once	4	(4.3)	88	(95.7)	**0.060**
Twice	1	(2.6)	37	(97.4)	
Three times	0	(.0)	30	(100.0)	
Four times	3	(18.8)	13	(81.3)	
More than four times	1	(6.7)	14	(93.3)	
**How long ago did the abuse take place**					
Days	6	(11.9)	37	(88.1)	**0.043**
Weeks	0	(.0)	4	(100.0)	
Months	3	(2.3)	126	(97.7)	
Years	0	(.0)	15	(100.0)	
**First person to tell of the abuse**					
Mother	5	(3.6)	145	(96.4)	**0.419**
Father	0	(.0)	8	(100.0)	
Guardian	0	(.0)	8	(100.0)	
Friend	1	(10.0)	10	(90.0)	
Teacher	3	(16.7)	11	(83.3)	
**How long before sharing**					
Same day of abuse	3	(2.8)	105	(97.2)	**0.022**
One day after the abuse	1	(2.2)	45	(97.8)	
One week after the abuse	2	(25.0)	8	(75.0)	
One month after the abuse	3	(11.1)	24	(88.9)	

To find out if there was any association between social demographic characteristics and sexual abuse profile. Bivariate analysis conducted indicated that PTSD was associated with perpetrator acts (p<0.0001), how long ago the abuse had taken place (p=0.043), how long before sharing the incidence (p=0.02).

**Table 3b:** Association between PTSD and sexual abuse profile at baseline (Bivariate Analysis).

	PTSD				
	Partial PTSD N=9		Full PTSD N=182		
	N	(%)	N	(%)	p-value
**First place to be taken**					
Hospital	6	(3.8)	155	(96.2)	**0.425**
Chief’s camp	0	(.0)	2	(100.0)	
Police station	3	(10.7)	25	(89.3)	
**How long before receiving medical attention**					
Within 1 hours	1	(.8)	124	(99.2)	**<0.0001**
Within 2 hours	0	(.0)	13	(100.0)	
Within 12 hours	2	(6.9)	27	(93.1)	
Within 48 hours	1	(20.0)	4	(80.0)	
Within 72 hours	0	(.0)	3	(100.0)	
After 72 hours	5	(31.3)	11	(68.8)	
**How did you cope with school work**					
Finishing homework	3	(2.3)	130	(97.7)	**0.093**
Not finishing homework	4	(7.4)	50	(92.6)	
**Attitude towards school changed**					
Yes	4	(2.3)	167	(97.7)	**0.001**
No	3	(18.8)	13	(81.3)	
**Used school to escape from abuse**					
Yes	4	(2.3)	167	(97.7)	**0.001**
No	3	(17.6)	14	(82.4)	

PTSD was associated with the length of time taken in receiving medical attention (p<0.0001), change of attitude towards school (p<0.001) and use of school to escape abuse (p<0.001).

**Table 4:** Predictors of Full PTSD at baseline (Multivariate Analysis Logistic Regression).

	B	S.E.	p-value	OR	95% C.I. for OR	
					Lower	Upper
Who do you live with	−0.663	0.857	**0.439**	0.515	0.096	2.762
What perpetrator made victim do	1.483	1.209	**0.22**	4.406	0.412	47.093
How long ago did the abuse take place	0.14	0.443	**0.752**	1.15	0.483	2.742
How long before sharing	0.273	0.46	**0.553**	1.314	0.533	3.237
How long before receiving medical attention	−0.734	0.36	**0.005**	0.48	0.237	0.972
Attitude towards school changed	−1.193	1.211	**0.325**	0.303	0.028	3.255
Used school to escape from abuse	0.643	1.367	**0.638**	1.902	0.13	27.737
Substance use	−1.265	0.686	**0.065**	0.282	0.074	1.084

When multivariate analysis was done, only the length of time taken to receive medical attention (p<0.005) was a predictor of PTSD.

**Table 5:** Educational status (below and above average) in relation to PTSD.

	Coefficient	S.E. of coefficient	p-value	OR	95% CI for OR	
					Lower	Upper
**Partial PTSD**	0.751	0.3	**0.01**	2.118	1.177	3.812
